# Effects of PTEN Loss and Activated KRAS Overexpression on Mechanical Properties of Breast Epithelial Cells

**DOI:** 10.3390/ijms19061613

**Published:** 2018-05-30

**Authors:** Will Linthicum, Minh-Tri Ho Thanh, Michele I. Vitolo, Qi Wen

**Affiliations:** 1 Biomedical Engineering Department, Worcester Polytechnic Institute, 100 Institute Road, Worcester, MA 01609, USA; whlinthicum@wpi.edu; 2 Physics Department, Worcester Polytechnic Institute, 100 Institute Road, Worcester, MA 01609, USA; htmtri@wpi.edu; 3 Greenebaum National Cancer Institute Comprehensive Cancer Center, University of Maryland School of Medicine, 655 West Baltimore St., Bressler Research Bldg., Rm. 10-039, Baltimore, MD 21201, USA

**Keywords:** atomic force microscopy, cancer, cell mechanics

## Abstract

It has previously been shown that the simultaneous activation of PI3K (phosphatidylinositol 3-kinase) and Ras/MAPK (mitogen-activated protein kinases) pathways facilitate tumor growth despite only inducing cancer cell dormancy individually. Determining the impacts on cellular mechanics each pathway incites alone and in unison is critical to developing non-toxic cancer therapies for triple-negative breast cancers. PTEN (phosphatase and tensin homolog) knockout and activated KRAS (Kristen rat sarcoma viral oncogene homolog) overexpression in healthy MCF-10A human breast epithelial cells activated the PI3K and Ras/MAPK pathways, respectively. Cell stiffness and fluidity were simultaneously measured using atomic force microscopy. Results suggest that PTEN knockout reduced cell stiffness and increased cell fluidity independent of PI3K activation. Effects of activated KRAS overexpression on cell stiffness depends on rigidity of cell culture substrate. Activated KRAS overexpression also counteracts the effects of PTEN knockout.

## 1. Introduction

Tumor initiation and progression are accompanied by dysregulation of certain signal pathways. Among the most frequently dysregulated pathways in cancer are the phosphatidylinositol 3-kinase (PI3K) pathway and the Ras/MAPK pathway [1]. Several forms of cancer, including prostate, lung, ovarian, colorectal, endometrial, melanoma, acute myeloid leukemia, and bladder, have increased activation of the PI3K and Ras/MAPK pathways [2,3,4,5,6,7,8,9]. Additionally, a large proportion of breast cancer, specifically triple negative breast cancer (TNBC), have a variety of mutations leading to the activation of both pathways [1]. Thompson et al. demonstrated that dual activation of the PI3K and Ras/MAPK pathways in non-tumorigenic MCF-10A breast cells via PTEN loss and KRAS activation promotes tumor initiation and growth [1]. However, individually activating either one was not sufficient to promote tumorigenesis, but generated a cell phenotype that behaves like dormant cancer cells [1], which are present in early tumor progression or in residuals left behind after the treatment of the primary tumor [10]. These dormant cancer cells can stay undetected in a quiescent state for long periods of time and can be reawakened years after the patients are deemed to be cured. Reliable detection of dormant cancer cells is critical to prevent cancer relapse.

Abnormal changes in cell mechanical properties are associated with the progression of cancer and many other diseases. Mechanical tests on cancer cell lines and cancer cells directly taken from patients suggest that cancer cells have a much lower value of elastic modulus than their normal counterparts [11,12,13,14]. It has also been reported that the stiffness of cancer cells is strongly correlated with the metastatic potential; the softer the cell, the higher the invasiveness [12,15]. Cell mechanical properties are determined mainly by the cytoskeleton, a complex network of actin filaments, intermediate filaments and microtubules [16]. These cytoskeletal filaments participate in important cellular functions including cell division, cell signaling, and motility. Many cancer drugs are targeted at modifying the organization of cytoskeletal components, especially the microtubules [17]. Many downstream targets of PTEN and KRAS have effects on structure and dynamics of the cytoskeletal components [18,19]. Therefore, PTEN and KRAS may play roles in regulating cell mechanical properties. Moreover, cell stiffness may also be a marker for detection of dormant cancer cells.

Atomic force microscopy (AFM) is an established technique to characterize mechanical properties of living cells [20]. To perform stiffness measurements, the atomic force microscope drives a soft cantilever to indent vertically into the cell. The cantilever position and the cantilever deflection due to the resistive force from the cell are measured with sub-nanometer resolution. Thus, allows precise measurements on the indentation force and the resulting cell deformation. Cell stiffness is extracted from the resulting force-indentation curve by fitting it to the Hertz model [21].

In this study, we report the impacts of PTEN loss and activated KRAS overexpression on mechanical properties of MCF-10A cells. We applied AFM to measure stiffness of the MCF-10A cell line and its mutant cells lines: the PTEN−/− cell line with PTEN deletion, the 10A-KRAS(G12V) cell line with overexpression of activated KRAS, and the PTEN−/−KRAS(G12V) cell line with both PTEN deletion and activated KRAS overexpression. We observed that PTEN loss and activated KRAS overexpression have differential effects on protein expression, actin cytoskeletal structure and mechanical properties of MCF-10A cells. 

## 2. Results

### 2.1. PTEN Knockout and KRAS Overexpression Modifies Actin Cytoskeleton Structure

**Figure 1:**
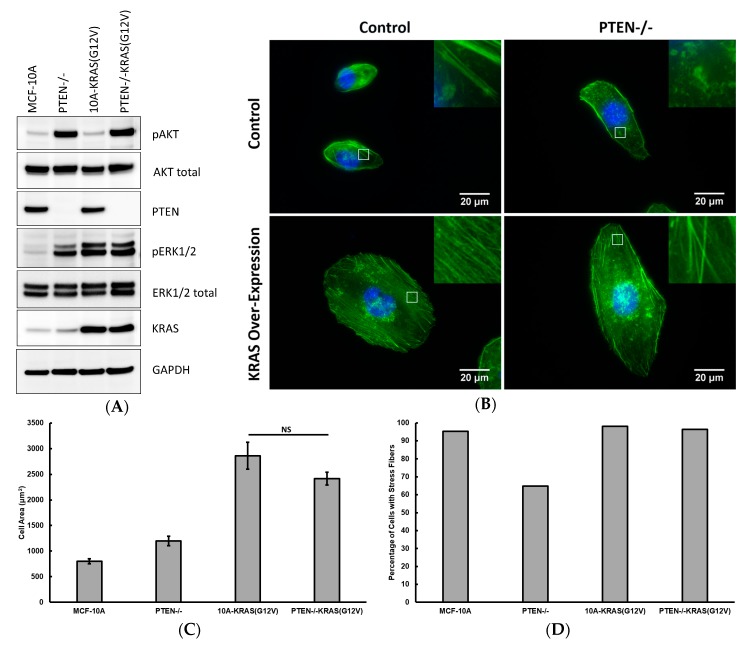
(**A**) Western blots of MCF-10A, PTEN−/−, 10A-KRAS(G12V), and PTEN−/−KRAS(G12V) cells highlighting proteins critical to the expression of the PI3K and Ras/MAPK pathways; (**B**) Immunofluorescence images of MCF-10A, PTEN−/−, 10A-KRAS(G12V), and PTEN−/−KRAS(G12V) cells stained with phalloidin (green) and DAPI (blue). Insets are zoomed views of the regions highlighted in the white boxes; (**C**) Average cell area and (**D**) percentage of cells with stress fibers for MCF-10A, PTEN−/−, 10A-KRAS(G12V), and PTEN−/−KRAS(G12V) cells. Number of cells measured: *n* = 50–63. NS signifies non-significant differences between two groups (*p* > 0.05).

Using the MCF-10A cells, which are non-tumorigenic, diploid mammary epithelial cells, provides a clean background of minimal mutations to determine phenotypic mechanical shifts due to specific, individual mutations. Western blot analysis (Figure 1A) shows the parental MCF-10A cells have minimal activation of both the PI3K and Ras/MAPK pathways since the phosphorylation of both AKT (protein kinase B) and ERK1/2 (extracellular signal-regulated kinases 1 and 2) are minimal. Upon PTEN deletion, activated AKT (pAKT) levels increased. Interestingly, although not a direct effector of PTEN loss, ERK1/2 activation (pERK1/2) also increased. This ERK activation is consistently observed in the PTEN−/− cell line [1,22] and may be attributed to pathway crosstalk and/or positive feedback loops [23]. Upon overexpression of the activated KRAS mutant (KRAS(G12V)), pERK1/2 levels increased without any increase in pAKT levels. Finally, the combination of PTEN loss and overexpression of activated KRAS yields an overactivation of both the PI3K and Ras/MAPK pathways, as seen by the high levels of pAKT and pERK1/2, respectively. Introduction of the mutations to activate each or both pathways also results in a variety of actin cytoskeleton compositions and overall cell morphologies, as shown in Figure 1B.

Quantitative results of cell area and percentage of cells of exhibiting stress fibers are shown in Figure 1C,D. Control MCF-10A cells are the smallest of the tested conditions and possess stress fibers in nearly all cells. PTEN loss alone results in slightly larger cells, with a decrease in the percentage of cells that express stress fibers. Both conditions with KRAS overexpression are at least three times larger than the control MCF-10A cells and continued to have a high probability of exhibiting stress fibers.

### 2.2. PTEN Knockout Reduces Cell Stiffness, Activated KRAS Overexpression Increases Cell Stiffness

**Figure 2:**
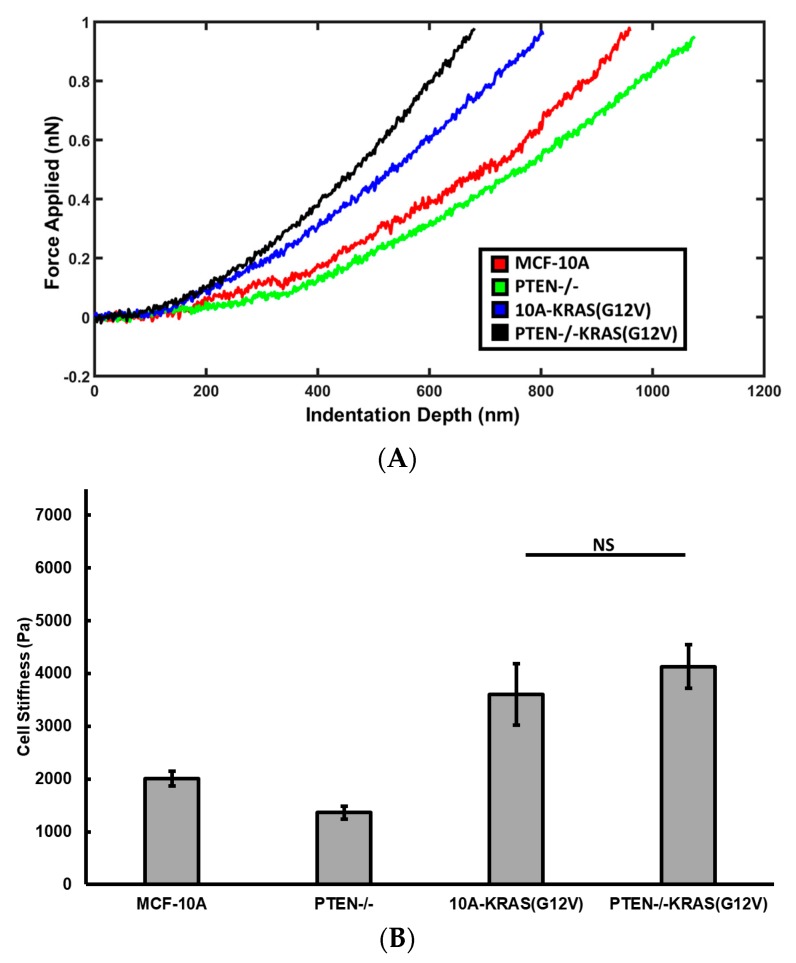
(**A**) Force curve examples that are representative of the average stiffness of MCF-10A, PTEN−/−, 10A-KRAS(G12V), and PTEN−/−KRAS(G12V) cells seeded on glass surface; (**B**) Average cell stiffness of cell seeded on glass surfaces. Number of cells measured: *n* = 16–35. NS signifies non-significant differences between two groups (*p* > 0.05).

Cells have varied stiffness values when transitioning between phenotypes and in response to their surrounding microenvironment [24,25]. Using atomic force microscopy, we measured the stiffness of the perinuclear region to gauge cell stiffness changes due to function of PTEN loss and activated KRAS overexpression. The stiffness of MCF-10A, PTEN−/−, 10A-KRAS(G12V), and PTEN−/−KRAS(G12V) cells seeded on collagen coated glass is shown in Figure 2.

PTEN−/− cells are significantly softer (*p* < 0.001) than the parental cell line MCF-10A. The knockout of PTEN results in reduced cell stiffness only when activated KRAS is not overexpressed. 10A-KRAS(G12V) cells are significantly stiffer than the control MCF-10A cells. Although PTEN−/−KRAS(G12V) cells are slightly stiffer than the 10A-KRAS(G12V) cells, the stiffness difference between these two cell lines is not statistically significant. These suggest that KRAS overexpression counteracts the effects of PTEN knockout on stiffness of PTEN−/−KRAS(G12V) cells.

### 2.3. PTEN Knockout and Activated KRAS Overexpression Affects Cell Fluidity

A cell can be modeled as a viscoelastic material. When subject to external force, it exhibits both elastic properties by resisting the force like a solid and viscous properties by flowing like a liquid. The viscoelasticity of a cell can be studied by imposing a small oscillatory deformation on the cell and measuring the force required to create such an oscillatory deformation. For a purely elastic material, the force and deformation are in phase, for a purely viscous material, the deformation lags force by a 90-degree phase lag. For a viscoelastic material, the phase lag is smaller than 90 degrees, and a larger phase lag means the material behaves more like a liquid. Therefore, the loss tangent, i.e., the tangent function of phase lag, is a measure of the cell fluidity.

**Figure 3:**
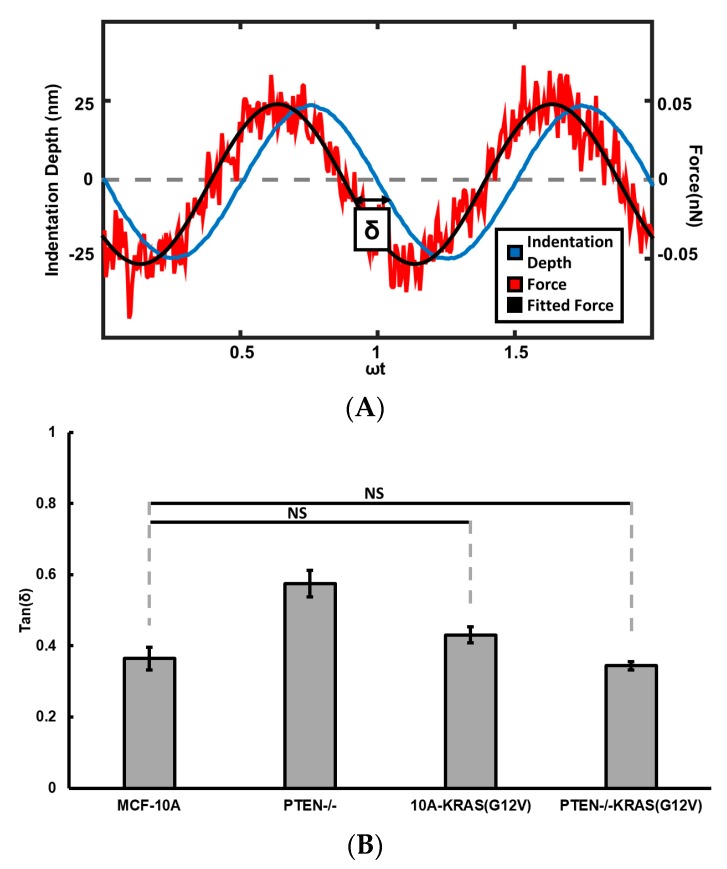
(**A**) Example oscillatory force (red) and indentation (blue) signals of a force curve, with phase shift δ depicted between the two signals. The force signal is fitted into a sinusoidal function of time as indicated by the black line. (**B**) Average loss tangent of control MCF-10A, PTEN−/−, 10A-KRAS(G12V), and PTEN−/−KRAS(G12V) cells seeded on glass. Number of cells measured: *n* = 16–35. NS signifies non-significant differences between two groups (*p* > 0.05).

We determined the fluidity of MCF-10A, PTEN−/−, 10A-KRAS(G12V), and PTEN−/−KRAS(G12V) cells seeded on glass using AFM by oscillating the cantilever at the lowest point of indentation, shown in Figure 3. Activated KRAS overexpression does not significantly change the fluidity, since loss tangent of MCF-10A cells is not significantly different from the loss tangents of 10A-KRAS(G12V). Knocking out PTEN in MCF-10A cells significantly increased cell fluidity, as the loss tangent of PTEN−/− cells is significantly larger than that of MCF-10A cells. However, the PTEN−/−KRAS(G12V) cells, with both PTEN loss and activated KRAS overexpression, have loss tangent values similar to that of MCF-10A cells. These suggest the activated Ras/MAPK pathway counteracts the effects of PTEN loss on cell viscoelasticity.

### 2.4. Effects of PTEN Knockout and Activated KRAS Overexpression Depends on Rigidity of Cell Culture Substrate

**Figure 4:**
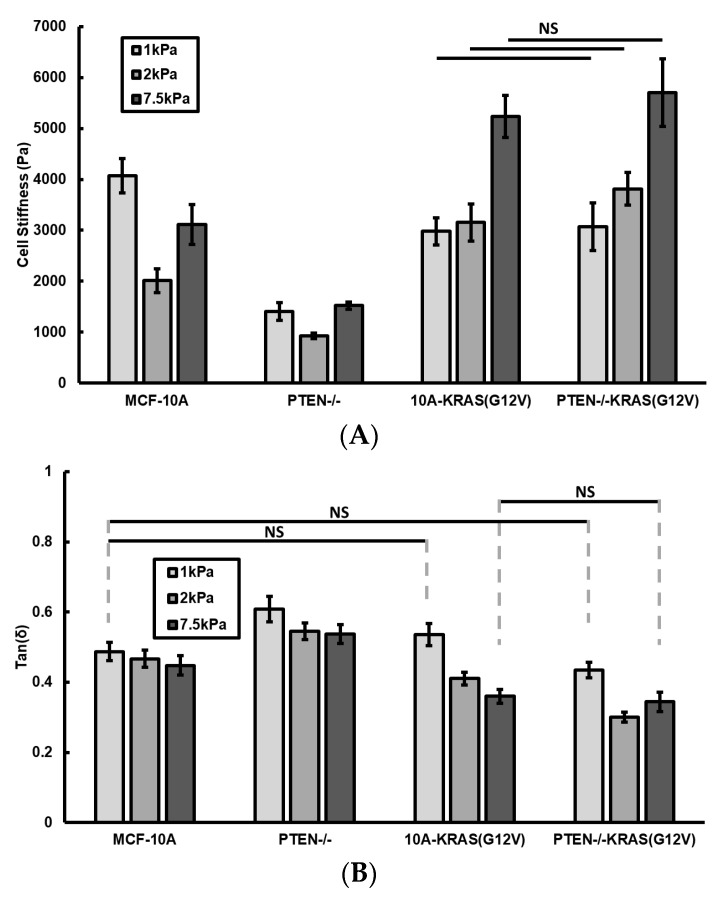
(**A**) Cell stiffness and (**B**) loss tangent (tan(δ)) values for control MCF-10A, PTEN−/−, 10A-KRAS(G12V), and PTEN−/−KRAS(G12V) cell conditions seeded on 1, 2, and 7.5 kPa collagen-coated polyacrylamide gels. Number of cells measured: *n* = 17–54. NS signifies non-significant differences between two groups (*p* > 0.05).

We also studied the effects of PTEN knockout and activated KRAS overexpression on the ability of cells to sense and adapt to the changes in extracellular matrix properties by measuring the cell stiffness and fluidity as functions of substrate rigidity. Results are shown in Figure 4A,B.

The two cell lines with activated KRAS overexpression responded similarly to substrate rigidity. Their stiffness increases as gel stiffness increases from 1 to 7.5 kPa. Unlike the two cell lines with KRAS overexpression, the PTEN−/− and MCF-10A cells both have stiffness values decrease when gel stiffness increases from 1 to 2 kPa, and then increase when gel stiffness increases from 2 to 7.5 kPa. These suggest that activated KRAS overexpression promotes cellular response to substrate rigidity, but PTEN loss does not.

On 1 kPa gel, the MCF-10A cells are stiffer than the three mutant cell lines. However, on 2 and 7.5 kPa gels, both the 10A-KRAS(G12V) cells and PTEN−/−KRAS(G12V) cells are stiffer than the MCF-10A cells. Cells seeded on 1 kPa gels followed the same viscoelastic behaviors trends as cells seeded on glass. Unlike on glass substrates, the MCF-10A cells had a higher loss tangent value than the 10A-KRAS(G12V) and PTEN−/−KRAS(G12V) cells when seeded on 2 and 7.5 kPa hydrogels. Additionally, there was no longer a significant decrease in loss tangent between the 10A-KRAS(G12V) and PTEN−/−KRAS(G12V) cells seeded on 7.5 kPa hydrogels. These indicate that substrate stiffness has differing effects on cellular viscoelastic behavior for cells containing different oncogenic mutations leading to the PTEN loss and/or MAPK pathways.

### 2.5. Effects of PI3K and ROCK Inhibition on PTEN Loss and Activated KRAS Overexpression Regulated Cell Stiffness

**Figure 5:**
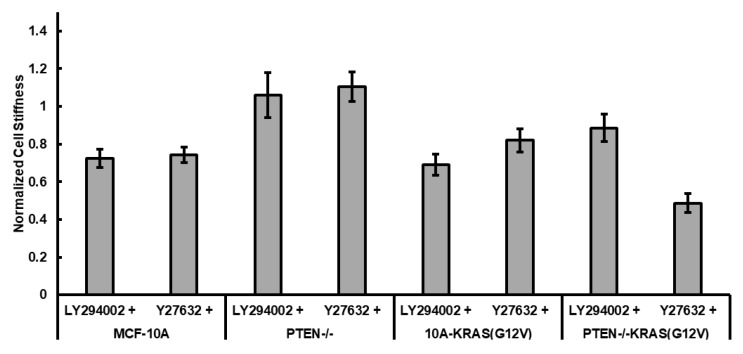
Effects of PI3K inhibitor and ROCK inhibitor on stiffness of MCF-10A, PTEN−/−, 10A-KRAS(G12V), and PTEN−/−KRAS(G12V) cells. Cells are seeded on collagen-coated substrates and treated with 20 µM LY294002 or 10 µM Y27632. Cell stiffness is normalized to the untreated cell stiffness of the same cell type. Number of cells measured: *n* = 14–33.

In order to study the specific mechanisms leading to changes in cytoskeletal stiffness as a result of PTEN loss and KRAS overexpression, we treated the cells with 20 µM LY294002, an inhibitor of PI3K signaling, and 10 µM Y27632, an inhibitor of rho-associated kinase (ROCK). Figure 5 shows the stiffness of treated cells normalized against their untreated controls. Both treatments slightly decreased the stiffness of MCF-10A cells. LY294002 treatment did not significantly affect the stiffness of PTEN−/− and PTEN−/−KRAS(G12V), indicating that activated PI3K in PTEN−/− cells does not account for the lower stiffness of PTEN−/− in comparison to MCF-10A. Although Y27632 treatment did not significantly change stiffness of 10A-KRAS(G12V) cells (*p* = 0.166), it caused a significant decrease in stiffness of PTEN−/−KRAS(G12V) cells (*p* = 0.003). This suggests that the combination of PTEN deletion and activated KRAS overexpression promotes a sensitivity to ROCK inhibition.

## 3. Discussion

Breast cancers have numerous mutations affecting a variety of different signaling pathways [26]. Among the most frequently dysregulated pathways in breast cancer are the phosphatidylinositol 3-kinase (PI3K) pathway and the Ras/MAPK pathway [2,27,28,29,30,31,32,33,34,35,36,37,38,39], and therefore we chose to manipulate one component of each pathway (i.e., PTEN deletion and KRAS(G12V) expression) to promote pathway activation. We used the MCF-10A cells as base cell line. The MCF-10A cells are non-tumorigenic, diploid, and genetically stable mammary epithelial cell line, which provide a clean background of minimal mutations to determine phenotypic mechanical shifts due to specific, individual mutations introduced. Additionally, the MCF-10A cells with added mutations begin to model triple-negative breast cancer, since these cells do not express the estrogen receptor (ER) or progesterone receptor (PR), nor do they overexpress Her2/Neu [1]. Due to the absence of ER, PR, and Her2/Neu overexpression, no targeted therapy exists for patients with triple negative breast cancer, and thus these patients must endure chemotherapeutic therapies with unpleasant toxic side-effects [40]. Once the effects of the individual mutations on cell stiffness are determined, we plan to incrementally combine oncogenic mutations, as we began to do in this study with the combination of PTEN loss and overexpression of the activated KRAS. Teasing out the effects of individual oncogenic mutations of cytoskeletal properties will aid in determining a more targeted and less toxic approach for cancer therapies to inhibit migration, invasion, circulating tumor cell survival and/or metastasis in general.

As a non-tumorigenic cell line, MCF-10A cells are stiffer than the tumorigenic breast cell lines. They are 1.4–1.8 times stiffer than the MCF7 cells, a transformed breast cancer cell line, and are about 4 times stiffer than MDA-MB-231 cells, a metastatic breast cancer cell line [41,42]. We presented in this work a first set of studies on the effects of PTEN loss and activated KRAS overexpression on cell mechanical properties of MCF-10A cells. As mutants of the same parental MCF-10A cell line, the PTEN−/−, the 10A-KRAS(G12V), and the PTEN−/−KRAS(G12V) cells are different from each other not only in protein expression but also in mechanical stiffness. Thompson et al. studied the behavior of these cells in vivo by injecting these cell lines into mice. They found that the PTEN−/−KRAS(G12V) caused robust tumor growth similar to that of the tumorigenic MDA-MB-231 breast cancer cells. Over time, approximately 50% of the mice injected with the 10A-KRAS(G12V) cells also form tumors (correspondence with M. Vitolo). However, the PTEN−/− are quiescent like dormant cancer cells. These cells persist in the mice for weeks longer than the MCF-10A parental counter-parts, but never grow to form tumors. Hence, our results suggest that cell stiffness measured by AFM can be a marker for detection of cancer cell lines with different mutations. 

Cell stiffness is regulated by actomyosin contractility and the structure of cytoskeleton. PI3K activation results in activation of AKT, which can regulate dynamics of actin cytoskeleton. Our results on LY294002 treatments suggest that increased pAKT level due to PI3K activation does not account for the reduced stiffness in PTEN−/− cells. A recent study by Vitolo et al. shows that although LY294002 inhibits the increase in pAKT in the PTEN−/− cells, it does not inactivate the overactivated cofilin [43], an actin-severing protein that destabilizes actin filaments. The activated cofilin in PTEN−/− cells may be the reason these cells are softer than the parental MCF-10A cells. In lieu of PI3K and cofilin activation, PTEN loss can control actin cytoskeleton through many other proteins.

Both PTEN loss and activated KRAS overexpression lead to increased level of ERK1/2 activation. However, PTEN−/− cells are softer and KRAS mutants are stiffer than parental MCF-10A cells. These suggest that levels of pERK1/2 may be important for cell mechanics, since PTEN−/− cells has lower ERK1/2 activation than cells with activated KRAS overexpression. Activated ERK1/2 leads to the activation of Rho-associated kinase (ROCK), which promotes actin polymerization, stress fiber formation and actin-myosin contraction [44]. Therefore, inhibiting ROCK could counter-act the effects of activated pERK1/2 on cell stiffness. Surprisingly, ROCK inhibition significantly decreased the stiffness for the double mutant PTEN−/−KRAS(G12V), but did not have a significant effect on the stiffness of 10A-KRAS(G12V) and PTEN−/− cells. Our observations call for further studies to identify the downstream molecules of PTEN loss and activated KRAS overexpression that regulate cell stiffness.

Our study has begun to analyze cytoskeletal changes due to introduced oncogenic mutations. Continued analysis of cytoskeletal alterations due to oncogenic mutations will enhance our understanding for the role of these changes in metastatic progression (i.e., migration, invasion, circulating tumor cell survival) to aid in more targeted therapeutic development to prevent metastasis.

## 4. Materials and Methods

### 4.1. Cell Lines and Cell Culture

MCF-10A cells were bought from ATCC (Manassas, VA, USA). PTEN−/−, 10A-KRAS(G12V) and PTEN−/−KRAS(G12V) cell lines were created from the MCF-10A cell line as previously described [1]. Cells were cultured at 37 °C and 5% CO_2_ in DMEM/F-12 media (Invitrogen, Grand Island, NY, USA) with 1% Penicillin-Streptomycin (Gemini Bio-Products, West Sacramento, CA, USA), 0.2 µg/mL recombinant human EGF (Invitrogen), 0.5 µg/mL hydrocortisone (Sigma, St. Louis, MO, USA), 0.2 µg/mL Cholera Toxin (Sigma), and 10 µg/mL Insulin (Sigma). PI3K and ROCK were inhibited by adding 20 mM LY294002 (Cell Signaling Technology, Danvers, MA, USA) or 10 mM Y27632 (Cell Signaling Technology, Danvers, MA, USA) in a 1:1000 volume ratio to cell culture dishes for 24 or 4–6 h before measurement respectively.

### 4.2. Collagen-Coated Surface Preparation

Polyacrylamide gels were made on (3-Aminopropyl) trimethoxysilane (APTMS) (Alfa Aesar, Haverhill, MA, USA) and glutaraldehyde (Amresco, Cleveland, OH, USA) treated 25 µm × 25 µm glass slides with acrylamide (Bio-Rad, Hercules, CA, USA), bis-acrylamide (Bio-Rad), 8.2 pH HEPES buffer (Amresco), ammonium persulfate (APS) (Amresco), and tetramethylethylenediamine (TEMED) (Amresco) as previously described [45]. Treated glass slides were UV-glued into culture dishes prior to gel preparation. Three volumes of acrylamide/bis-acrylamide (5%/0.04%, 5%/0.08%, 8%/0.10%) were used to obtain three surface stiffness conditions of 1, 2, and 7.5 kPa respectively. The gels were coated with 0.1 mg/mL type I rat tail collagen after the gels were UV treated with 1 mg/mL sulfo-SANPAH (ProteoChem, Loves Park, IL, USA). Similarly, glass slides were coated with 0.1 mg/mL type I rat tail collagen after being incubated at room temperature in 0.1 mg/mL Poly-l-Lysine. Cells were seeded onto the surfaces at 10% confluency prior to measurement.

### 4.3. Western Blotting

Cells (2 × 10^6^ per 100 mm dish) were seeded in DMEM/F12 supplemented with 1% charcoal/dextraned stripped FBS (Hyclone, South Logan, UT, USA) and 1% Penicillin-Streptomycin and harvested for lysis after 48 h. Growth in this minimal media was required to remove exogenous growth factors and allow for only basal activation of signaling pathways. Cells were harvested in immunoprecipitation lysis buffer [0.5 mol/L Tris-HCl, pH 7.4, 1.5 mol/L NaCl, 2.5% deoxycholic acid, 10% NP-40, 10 mmol/L EDTA] supplemented with protease inhibitor cocktail EDTA-free (Roche, Mannheim, Germany) and phosphatase inhibitor cocktail II (Calbiochem, La Jolla, CA, USA). Western blotting was performed with 4–12% NuPage Bis-Tris gels (Invitrogen) in MES running buffer. Proteins were transferred to PDVF membranes. Primary antibodies anti-p-AKT(S473), anti-AKT, anti-p-ERK1/2, anti-ERK1/2, anti-PTEN (Cell Signaling, Danvers, MA, USA), anti-KRAS (EMD Millipore, Billerica, MA, USA) and anti-GAPDH (Abcam, Cambridge, MA, USA) were used at the manufacturers’ recommended dilutions.

### 4.4. Immunofluorescence

Cells seeded for 24 h on glass were washed with 37 °C PBS+ (supplemented with 1 mM Ca^2+^ and 0.5 mM Mg^2+^), fixed with 3.7% paraformaldehyde in PBS for 10 min, then permeabilized in 0.1% Triton-X PBS (Alfa Aesar, Haverhill, MA, USA) for 15 min. To visualize actin organization, cells were stained with Alexa Fluor488 Phalloidin (Molecular Probes, Eugene, OR, USA) at 4 units per 1 mL PBS for 30 min, followed by nucleus staining with 1 μg/mL DAPI (Biotium, Fremont, CA, USA) for 10 min. Fluorescent images were captured using a QIClick Camera (QImaging, BC, Canada) mounted on an Olympus IX83 microscope (Olympus America Inc., Center Valley, PA, USA).

### 4.5. AFM Stiffness and Viscoelasticity Assay

All measurements were performed utilizing an MFP-3D-BIO atomic force microscope (Asylum Research, Santa Barbara, CA, USA) and DNP cantilevers (Bruker, Camarillo, CA, USA) with nominal spring constant 0.06 N/m. To ensure reliability of measurements, each cantilever was calibrated for spring constant (k) through thermal tuning, and for inverse optical lever sensitivity (InvOLS) through linear force curve fitting on glass in liquid.

Live cells were measured 24 h after seeding on the desired surfaces to ensure proper spreading, while preventing high confluency. Measurements were limited to isolated cells to reduce the influence of cell-cell communication. Phase contrast microscopy was used in unison with AFM to align the cantilever tip over desired measurement areas of the cell. Individual force curves were taken in three locations in the perinuclear region of each cell to guarantee the thickness of the cell was significantly greater than the distance the cantilever indented into the cell. Each force curve was taken at a velocity of 2 µm/s and to a trigger point of 1 nN. 15–20 cells were measured per dish and no longer than 30 min after the dish was removed from the incubator to ensure cell viability. 

Using a custom MATLAB code, cantilever deflection as a function of sample indentation depth was extracted from AFM force curves, with examples shown in Figure 2A. Stiffness values were determined from the deflection-indentation curve using the Hertz model with a conical tip:

(1) $$E=\frac{kd\pi \left(1-\nu^{2}\right)}{2\Delta^{2}\tan\phi }$$

where k is the cantilever spring constant, d is the cantilever deflection, ν is the Poisson’s ratio value (using 0.5), Δ is the sample indentation depth, and ϕ is half the conical opening angle of the AFM tip (here, 35°) [20]. To minimize the effects of nonlinear effects, force-indentation curves were fit to the Hertz model over the first 400 nm indentation depth.

### 4.6. Viscoelasticity Measurements by AFM

To measure the cell fluidity simultaneously with the stiffness measurements, we impose to the AFM cantilever a small amplitude sinusoidal oscillatory motion, 25 nm in amplitude and 10 Hz in frequency, when it reaches the deepest point of indentation. The resulting oscillatory force and cell deformation were recorded as shown in Figure 2B. The phase lag between cell deformation and force is determined and the tangent function of phase lag, i.e., loss tangent is reported to represent the fluidity of cells.

## Abbreviations

PI3K	Phosphatidylinositol 3-kinase
MAPK	Mitogen-activated protein kinases
PTEN	Phosphatase and tensin homolog
AFM	Atomic force microscope
ROCK	Rho-associated kinase

## References

[1] Thompson K.N., Whipple R.A., Yoon J.R., Lipsky M., Charpentier M.S., Boggs A.E., Chakrabarti K.R., Bhandary L., Hessler L.K., Martin S.S. (2015). The combinatorial activation of the PI3K and Ras/MAPK pathways is sufficient for aggressive tumor formation, while individual pathway activation supports cell persistence. Oncotarget.

[2] Cancer Genome Atlas Network (2012). Comprehensive molecular portraits of human breast tumours. Nature.

[3] Kinkade C.W., Castillo-Martin M., Puzio-Kuter A., Yan J., Foster T.H., Gao H., Sun Y., Ouyang X., Gerald W.L., Cordon-Cardo C. (2008). Targeting AKT/mTOR and ERK MAPK signaling inhibits hormone-refractory prostate cancer in a preclinical mouse model. J. Clin. Investig..

[4] Downward J. (2008). Targeting RAS and PI3K in lung cancer. Nat. Med..

[5] De Roock W., Claes B., Bernasconi D., De Schutter J., Biesmans B., Fountzilas G., Kalogeras K.T., Kotoula V., Papamichael D., Laurent-Puig P. (2010). Effects of KRAS, BRAF, NRAS, and PIK3CA mutations on the efficacy of cetuximab plus chemotherapy in chemotherapy-refractory metastatic colorectal cancer: A retrospective consortium analysis. Lancet Oncol..

[6] Ihle N.T., Lemos R., Wipf P., Yacoub A., Mitchell C., Siwak D., Mills G.B., Dent P., Kirkpatrick D.L., Powis G. (2009). Mutations in the phosphatidylinositol-3-kinase pathway predict for antitumor activity of the inhibitor PX-866 whereas oncogenic Ras is a dominant predictor for resistance. Cancer Res..

[7] Steelman L.S., Chappell W.H., Abrams S.L., Kempf R.C., Long J., Laidler P., Mijatovic S., Maksimovic-Ivanic D., Stivala F., Mazzarino M.C. (2011). Roles of the Raf/MEK/ERK and PI3K/PTEN/Akt/mTOR pathways in controlling growth and sensitivity to therapy-implications for cancer and aging. Aging.

[8] Shimizu T., Tolcher A.W., Papadopoulos K.P., Beeram M., Rasco D.W., Smith L.S., Gunn S., Smetzer L., Mays T.A., Kaiser B. (2012). The clinical effect of the dual-targeting strategy involving PI3K/AKT/mTOR and RAS/MEK/ERK pathways in patients with advanced cancer. Clin. Cancer Res..

[9] Knowles M.A., Hurst C.D. (2015). Molecular biology of bladder cancer: New insights into pathogenesis and clinical diversity. Nat. Rev. Cancer.

[10] Aguirre-Ghiso J.A. (2006). The problem of cancer dormancy: Understanding the basic mechanisms and identifying therapeutic opportunities. Cell Cycle.

[11] Hayashi K., Iwata M. (2015). Stiffness of cancer cells measured with an AFM indentation method. J. Mech. Behav. Biomed. Mater..

[12] Xu W.W., Mezencev R., Kim B., Wang L.J., McDonald J., Sulchek T. (2012). Cell Stiffness Is a Biomarker of the Metastatic Potential of Ovarian Cancer Cells. PLoS ONE.

[13] Hou H.W., Li Q.S., Lee G.Y.H., Kumar A.P., Ong C.N., Lim C.T. (2009). Deformability study of breast cancer cells using microfluidics. Biomed. Microdevices.

[14] Cross S.E., Jin Y.S., Rao J., Gimzewski J.K. (2007). Nanomechanical analysis of cells from cancer patients. Nat. Nanotechnol..

[15] Tavares S., Vieira A.F., Taubenberger A.V., Araujo M., Martins N.P., Bras-Pereira C., Polonia A., Herbig M., Barreto C., Otto O. (2017). Actin stress fiber organization promotes cell stiffening and proliferation of pre-invasive breast cancer cells. Nat. Commun..

[16] Pegoraro A.F., Janmey P., Weitz D.A. (2017). Mechanical Properties of the Cytoskeleton and Cells. Cold Spring Harb. Perspect. Biol..

[17] Jordan M.A., Wilson L. (1998). Microtubules and actin filaments: Dynamic targets for cancer chemotherapy. Curr. Opin. Cell Biol..

[18] Bhattacharya K., Maiti S., Mandal C. (2016). PTEN negatively regulates mTORC2 formation and signaling in grade IV glioma via Rictor hyperphosphorylation at Thr1135 and direct the mode of action of an mTORC1/2 inhibitor. Oncogenesis.

[19] Choi C., Helfman D.M. (2014). The Ras-ERK pathway modulates cytoskeleton organization, cell motility and lung metastasis signature genes in MDA-MB-231 LM2. Oncogene.

[20] Thomas G., Burnham N.A., Camesano T.A., Wen Q. (2013). Measuring the mechanical properties of living cells using atomic force microscopy. J. Vis. Exp..

[21] Hertz H. (1881). Über die Berührung fester elastischer Körper. J. Reine Angew. Math..

[22] Vitolo M.I., Weiss M.B., Szmacinski M., Tahir K., Waldman T., Park B.H., Martin S.S., Weber D.J., Bachman K.E. (2009). Deletion of PTEN promotes tumorigenic signaling, resistance to anoikis, and altered response to chemotherapeutic agents in human mammary epithelial cells. Cancer Res..

[23] Mendoza M.C., Er E.E., Blenis J. (2011). The Ras-ERK and PI3K-mTOR pathways: Cross-talk and compensation. Trends Biochem. Sci..

[24] Lin H.H., Lin H.K., Lin I.H., Chiou Y.W., Chen H.W., Liu C.Y., Harn H.I., Chiu W.T., Wang Y.K., Shen M.R. (2015). Mechanical phenotype of cancer cells: Cell softening and loss of stiffness sensing. Oncotarget.

[25] Park J.S., Chu J.S., Tsou A.D., Diop R., Tang Z.Y., Wang A.J., Li S. (2011). The effect of matrix stiffness on the differentiation of mesenchymal stem cells in response to TGF-beta. Biomaterials.

[26] Hanahan D., Weinberg R.A. (2011). Hallmarks of cancer: The next generation. Cell.

[27] Adeyinka A., Nui Y., Cherlet T., Snell L., Watson P.H., Murphy L.C. (2002). Activated mitogen-activated protein kinase expression during human breast tumorigenesis and breast cancer progression. Clin. Cancer Res..

[28] Britten C.D. (2013). PI3K and MEK inhibitor combinations: Examining the evidence in selected tumor types. Cancer Chemother. Pharmacol..

[29] Davis N.M., Sokolosky M., Stadelman K., Abrams S.L., Libra M., Candido S., Nicoletti F., Polesel J., Maestro R., D’Assoro A. (2014). Deregulation of the EGFR/PI3K/PTEN/Akt/mTORC1 pathway in breast cancer: Possibilities for therapeutic intervention. Oncotarget.

[30] Hanahan D., Weinberg R.A. (2000). The hallmarks of cancer. Cell.

[31] Hoeflich K.P., O’Brien C., Boyd Z., Cavet G., Guerrero S., Jung K., Januario T., Savage H., Punnoose E., Truong T. (2009). In vivo antitumor activity of MEK and phosphatidylinositol 3-kinase inhibitors in basal-like breast cancer models. Clin. Cancer Res..

[32] Mirzoeva O.K., Das D., Heiser L.M., Bhattacharya S., Siwak D., Gendelman R., Bayani N., Wang N.J., Neve R.M., Guan Y. (2009). Basal subtype and MAPK/ERK kinase (MEK)-phosphoinositide 3-kinase feedback signaling determine susceptibility of breast cancer cells to MEK inhibition. Cancer Res..

[33] Pereira C.B., Leal M.F., de Souza C.R., Montenegro R.C., Rey J.A., Carvalho A.A., Assumpcao P.P., Khayat A.S., Pinto G.R., Demachki S. (2013). Prognostic and predictive significance of MYC and KRAS alterations in breast cancer from women treated with neoadjuvant chemotherapy. PLoS ONE.

[34] Roberts P.J., Usary J.E., Darr D.B., Dillon P.M., Pfefferle A.D., Whittle M.C., Duncan J.S., Johnson S.M., Combest A.J., Jin J. (2012). Combined PI3K/mTOR and MEK inhibition provides broad antitumor activity in faithful murine cancer models. Clin. Cancer Res..

[35] Saini K.S., Loi S., de Azambuja E., Metzger-Filho O., Saini M.L., Ignatiadis M., Dancey J.E., Piccart-Gebhart M.J. (2013). Targeting the PI3K/AKT/mTOR and Raf/MEK/ERK pathways in the treatment of breast cancer. Cancer Treat. Rev..

[36] Sanchez-Munoz A., Gallego E., de Luque V., Perez-Rivas L.G., Vicioso L., Ribelles N., Lozano J., Alba E. (2010). Lack of evidence for KRAS oncogenic mutations in triple-negative breast cancer. BMC Cancer.

[37] Shah S.P., Roth A., Goya R., Oloumi A., Ha G., Zhao Y., Turashvili G., Ding J., Tse K., Haffari G. (2012). The clonal and mutational evolution spectrum of primary triple-negative breast cancers. Nature.

[38] Sivaraman V.S., Wang H., Nuovo G.J., Malbon C.C. (1997). Hyperexpression of mitogen-activated protein kinase in human breast cancer. J. Clin. Investig..

[39] Lopez-Knowles E., O’Toole S.A., McNeil C.M., Millar E.K.A., Qiu M.R., Crea P., Daly R.J., Musgrove E.A., Sutherland R.L. (2010). PI3K pathway activation in breast cancer is associated with the basal-like phenotype and cancer-specific mortality. Int. J. Cancer.

[40] Reis-Filho J.S., Tutt A.N. (2008). Triple negative tumours: A critical review. Histopathology.

[41] Li Q.S., Lee G.Y., Ong C.N., Lim C.T. (2008). AFM indentation study of breast cancer cells. Biochem. Biophys. Res. Commun..

[42] Agus D.B., Alexander J.F., Arap W., Ashili S., Aslan J.E., Austin R.H., Backman V., Bethel K.J., Bonneau R., Physical Sciences-Oncology Centers Network (2013). A physical sciences network characterization of non-tumorigenic and metastatic cells. Sci. Rep..

[43] Vitolo M.I., Boggs A.E., Whipple R.A., Yoon J.R., Thompson K., Matrone M.A., Cho E.H., Balzer E.M., Martin S.S. (2013). Loss of PTEN induces microtentacles through PI3K-independent activation of cofilin. Oncogene.

[44] Tong J., Li L., Ballermann B., Wang Z. (2016). Phosphorylation and Activation of RhoA by ERK in Response to Epidermal Growth Factor Stimulation. PLoS ONE.

[45] Menter P. (2000). Acrylamide Polymerization—A Practical Approach.

